# Lipid droplets: New roles as mediators of biotic and abiotic stress

**DOI:** 10.1093/plphys/kiae340

**Published:** 2024-06-18

**Authors:** Hannah M McMillan

**Affiliations:** Assistant Features Editor, Plant Physiology, American Society of Plant Biologists; Department of Biology, Duke University, Durham, NC 27708, USA

In an ever-changing environment, plants must respond to a wide array of abiotic and biotic stress. Many studies describe transcriptomic and proteomic changes in response to stress and even unveil functional implications of these changes (Kumar et al. 2016; Zhu 2016; Crawford et al. 2018). While less well-studied, lipidomic changes also play critical roles in plant responses to abiotic and biotic stress (Niu and Xiang 2018). For example, changing membrane lipid composition and modifications can contribute to a more appropriate barrier against stress, and lipid remodeling mechanisms can also allow cells to restructure the protein content of their internal and external membranes (Niu and Xiang 2018). In this issue of *Plant Physiology*, Scholz et al. describe protein composition and lipid modification changes in response to fungal and bacterial challenge as well as heat stress in Arabidopsis leaves (Scholz et al. 2024). Their results suggest an intriguing potential role for lipid droplets (LDs) in stress-related membrane remodeling and reveal a previously uncharacterized interplay between lipid remodeling mechanisms and defense hormone regulation.

**Figure 1.**
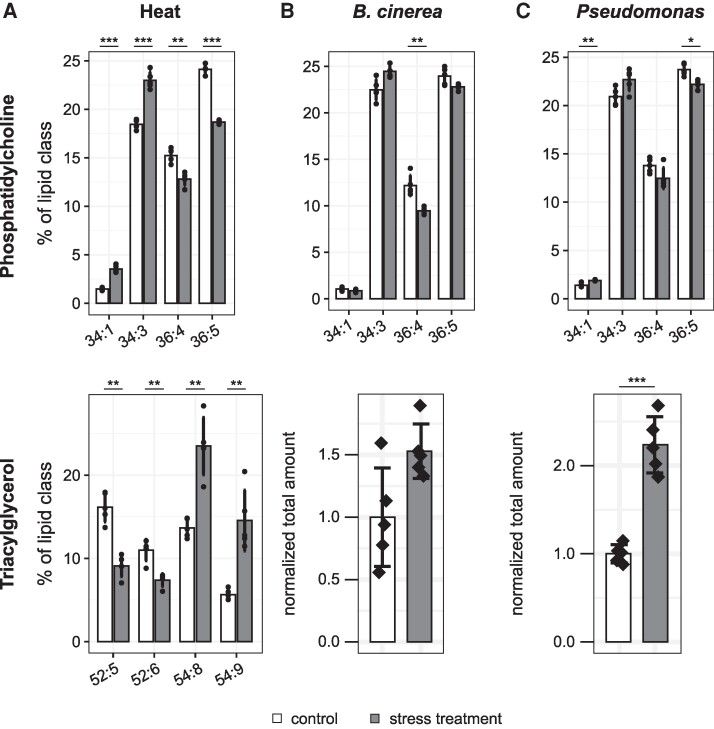
Changes in abundance of phosphatidylcholine and triacylglycerol species under 3 stress conditions. A–C) Lipidomic analysis of 7-week-old Arabidopsis plants after **(A)** 24-h heat stress at 37 °C, **(B)** spray inoculation with *Botrytis cinerea*, or **(C)** spray inoculation with *Pseudomonas syringae* pv. tomato DC3000 *ΔavrPto/ΔavrPtoB.* Statistics: Student's *t* test with Holm-Bonferroni correction. Values: Mean ± standard deviation. * and *** for *P* < 0.05 and *P* < 0.001, respectively. Figure modified from Scholz et al. (2024).

Interest in LDs has surged due to recent advances in our understanding of their diverse functional capabilities. LDs consist of a hydrophobic core of neutral lipids surrounded by a monolayer of phospholipids with embedded proteins (Guzha et al. 2023). Much of the LD literature to date focuses on the role LDs play in lipid storage, particularly in oilseeds (Bouchnak et al. 2023). However, Scholz et al. propose that during stress, LDs may serve as crucial sinks for acyl chains removed from the plasma and plastid membranes and sources for free sterols or sterol derivatives (Scholz et al. 2024). The LD core is comprised mainly of triacylglycerols and sterol esters (Guzha et al. 2023). During heat stress, cells replace unsaturated acyl chains containing more double bonds with saturated acyl chains containing fewer double bonds, which could contribute to increased membrane fluidity (Niu and Xiang 2018). As expected, in this study membrane lipids with more unsaturated acyl chains are replaced with membrane lipids containing more saturated acyl chains during heat stress (Scholz et al. 2024). For example, 34:1 and 34:3 phosphatidylcholine species increase, while 36:4 and 36:5 species decrease (Fig. 1A) (Scholz et al. 2024). Simultaneously and in contrast, 54:8 and 54:9 triacylglycerols (relatively unsaturated species) increase, while 52:5 and 52:6 triacylglycerols (relatively saturated species) decrease (Fig. 1A) (Scholz et al. 2024). The simultaneous decrease in unsaturated membrane lipids and increase in triacylglycerols could suggest that LDs absorb the discarded unsaturated acyl chains from membrane lipids in triacylglycerols to facilitate membrane remodeling.

In response to pathogen infection, a different trend was observed. While *Botrytis cinerea* and avirulent *Pseudomonas syringae* infection still led to modest, though not significant, increases in 34:3 phosphatidylcholine species and sometimes 34:1 species and decreases in 36:4 species, the unsaturated 36:6 phosphatidylcholine species was significantly increased in response to both pathogens (Fig. 1B and C) (Scholz et al. 2024). Similar to heat stress, triacylglycerols increased in response to both pathogens (Fig. 1B and C), mainly as a result of increases in the unsaturated 54:8 and 54:9 species (Scholz et al. 2024). These trends could suggest that LDs serve similar roles during pathogen infection and heat stress as sinks for unsaturated fatty acids, although the functional implications for membrane dynamics caused by substituting more saturated acyl chains remain unclear.

To determine if LD proteins may contribute to LD roles in heat and pathogen stress, Scholz et al. used the Arabidopsis double mutant *tgd1-1 sdp1-4*, which overaccumalates LDs and triacylglycerols (Scholz et al. 2024). As expected, basal levels of triacylglycerols increased in the double mutant, and membrane lipids contained lower levels of unsaturated acyl chains. Interestingly, the most unsaturated 54:9 triacylglycerol species showed lower basal levels in the double mutant unlike was seen in the 3 stress conditions where LD number and the 54:9 species increased. Even more intriguing, under stressed and unstressed conditions, the double mutant showed higher levels of the defense response proteins PR2, PR3, and PR5 and higher levels of the defense hormone salicylic acid compared with wild-type plants. A simultaneous decrease was observed in the allene oxide cyclase proteins AOC2 and AOC4, which are involved in biosynthesis of the antagonistic jasmonic acid plant hormone. The unexpected increase in defense hormone level in the LD-accumulating double mutant reveals an unexpected link between LD accumulation and plant hormone regulation.

Overall, this study presents an important step forward in understanding how cells coordinate protein and lipid changes to adapt to both biotic and abiotic stress. It seems that cells may restructure their membranes similarly during heat stress and responses to pathogens, favoring plasma and plastid membrane phospholipids that contain more saturated acyl chains, and that cells may utilize LDs to facilitate this remodeling. Indeed, the underlying reasons for why a similar restructuring occurs during abiotic and biotic stresses may even be similar. Salicylic acid plays well-documented roles in defense against pathogens by activating signaling pathways designed to contain and eliminate the biotic threat (Spoel and Dong 2024). Salicylic acid may also play a role in plant responses to temperature, as mutants deficient in salicylic acid pathways show decreased thermotolerance (Larkindale et al. 2005, Niu and Xiang 2018). Further, changing the saturation of certain lipid species restores salicylic and jasmonic acid signaling in some Arabidopsis mutants, linking regulation of lipid saturation with hormone signaling (Kachroo et al. 2004; Niu and Xiang 2018). While the trend toward saturated lipids in this study (Scholz et al. 2024) differs from other links between lipid remodeling and hormone signaling (Kachroo et al. 2004), further studies may reveal intricacies in lipid remodeling and LD-mediated signaling that help clarify their role in complex plant stress responses.

Although the role hormones play may differ in responses to abiotic and biotic stress, this study shows that membrane remodeling and LDs may contribute significantly to hormone regulation and signaling (Scholz et al. 2024). Given the similarities between abiotic and biotic stresses revealed here, it is interesting to consider how these discoveries may contribute to our understanding of how plants respond to combined stresses. Future work will be greatly beneficial to improve our understanding of these complex processes and reveal potential molecular targets to improve plant performance in a changing climate.
